# Reimagining Global Oncology Clinical Trials for the Postpandemic Era: A Call to Arms

**DOI:** 10.1200/GO.20.00346

**Published:** 2020-09-08

**Authors:** Kamal S. Saini, Begoña de las Heras, Ruth Plummer, Victor Moreno, Marco Romano, Javier de Castro, Philippe Aftimos, Judy Fredriksson, Gouri Shankar Bhattacharyya, Martin Sebastian Olivo, Gaia Schiavon, Kevin Punie, Jesus Garcia-Foncillas, Ernesto Rogata, Richie Pfeiffer, Cecilia Orbegoso, Kenneth Morrison, Giuseppe Curigliano, Lynda Chin, Monika Lamba Saini, Øystein Rekdal, Steven Anderson, Javier Cortes, Manuela Leone, Janet Dancey, Chris Twelves, Ahmad Awada

**Affiliations:** ^1^ Covance, Princeton, NJ; ^2^ East Suffolk and North Essex NHS Foundation Trust, Ipswich, United Kingdom; ^3^ Madrid Medical Doctors Association, Madrid, Spain; ^4^ Translational and Clinical Research Institute, Newcastle University, Newcastle-upon-Tyne, United Kingdom; ^5^ START Madrid-FJD, Hospital Fundación Jiménez Díaz, Madrid, Spain; ^6^ Hospital Universitario La Paz, IdiPAZ, Madrid, Spain; ^7^ Oncology Medicine Department, Institut Jules Bordet, Université Libre de Bruxelles, Brussels, Belgium; ^8^ F Hoffmann-La Roche, Basel, Switzerland; ^9^ Salt Lake City Medical Centre, Kolkata, India; ^10^ Immunomedics, Morris Plains, NJ; ^11^ R&D Oncology, AstraZeneca, Cambridge, United Kingdom; ^12^ Department of General Medical Oncology and Multidisciplinary Breast Centre, Leuven Cancer Institute, University Hospitals Leuven, Leuven, Belgium; ^13^ University Hospital Fundacion Jimenez Diaz, Autonomous University of Madrid, Madrid, Spain; ^14^ Leeds Cancer Centre, Patient and Public Involvement Group, Leeds, United Kingdom; ^15^ Istituto Europeo di Oncologia, IRCCS, Milan, Italy; ^16^ University of Milano, Milan, Italy; ^17^ Apricity Health, Houston, TX; ^18^ Dell Medical School at the University of Texas at Austin, Austin, TX; ^19^ HistoGeneX, Antwerp, Belgium; ^20^ Lytix Biopharma, Oslo, Norway; ^21^ IOB Institute of Oncology, Quiron Group, Madrid, Spain; ^22^ Canadian Cancer Trials Group, Queen’s University, Kingston, Ontario, Canada; ^23^ University of Leeds and Leeds Teaching Hospitals Trust, Leeds, United Kingdom

## INTRODUCTION

The process of developing new anticancer therapeutics has been considered by some to be expensive,^1^ time consuming,^2^ bureaucratic,^3^ and, to some extent, inefficient.^4^ The coronavirus disease 2019 (COVID-19) pandemic has significantly affected clinical oncology studies^5,6^ and underlined the need to embrace and accelerate long-pending and awaited reforms to cancer clinical trial methodology.^7-9^

This article highlights the need for optimal use of technology, reduced paperwork and bureaucracy, speedier trial setup, and greater patient centricity in the design and conduct of future clinical and translational cancer studies around the world.

## INCREASED USE OF TECHNOLOGY

The basic technology to enable secure and reliable telephone/video contact between clinicians, study coordinators, and patients to facilitate remote medical consultation has been available for a number of years; however, its adoption has been limited for a variety of reasons, including lack of access to such technologies in some developing countries and concerns surrounding privacy, safety, financial reimbursement, and legal and regulatory issues. Changes to reimbursement rules and regulations have been recently announced to encourage the use of telemedicine during the COVID-19 lockdown.^10,11^ These and other such pandemic-era reforms should be adopted not only for the current situation but should also be considered for permanent adoption.^12-14^

Electronic consent and telemedicine consultation could replace some protocol-mandated clinic visits, especially those for which medical imaging, biosample collection, or physical examination are not required.^15^ Reduced exposure to the hospital environment could enhance patient safety, comfort, and quality of life while also perhaps lowering the number of protocol deviations in clinical trials and the overall burden of trial participation.^16^ It may also help reduce inequalities in access to clinical trials resulting from transportation challenges because of geographic, financial, or physical issues, and would also reduce the burden on patient caregivers. Virtual formats could replace in-person investigator meetings, steering committee meetings, etc, thereby reducing physical, financial, and environmental burden while increasing speed and flexibility. Using social media as an easily accessible communication tool could also help to optimize the care of patients enrolled in cancer clinical trials.^17^ Measures that can be self-reported by patients are increasingly being used in clinical practice and trials, and these data can be collected remotely as electronic patient-reported outcomes (ePROs).

During the ongoing pandemic, patients, clinicians, and hospitals have become increasingly comfortable using telemedicine, and many stakeholders agree that the resulting improved access, lower cost, reduced risk of infection, and time saved should make telemedicine an integral part of our standard practice in cancer care and research moving forward, at least for predefined activities.^18,19^ Adopting digital pathology and radiomics platforms to enable images to be seamlessly analyzed at a remote and/or central facility could increase efficiency in cancer trials.^20,21^

Use of artificial intelligence could help efficiently match the unique clinical and molecular pathology characteristics of a given patient to relevant clinical trials within their region of the country,^22,23^ thereby providing access to potentially life-enhancing trials for a broader and more diverse population.^24^

Physical activity trackers, smart watches and other wearable devices, and smartphones with health applications allow for the real-time remote collection of health parameters, such as physical activity, ECG, temperature, blood glucose level,^25^ oxygen saturation,^26^ and ePROs.^27^ Validated, secure, and approved wearable devices could autopopulate trial databases with robust longitudinal data, reducing the need for manual data entry and providing more efficient and improved data quality and integrity.^28^ Use of clinical decision support algorithms to detect early signs and symptoms of concern (eg, fever and tachycardia suggestive of infection) and to alert the treating clinical team should be explored in cancer clinical trials where feasible.^29^ Such algorithms can also be customized to support specific clinical trial protocols, which may help to improve protocol adherence and care consistency across investigators in decentralized sites so that clinical trials can be opened in additional community sites and be more accessible for more patients closer to their homes.

Remote monitoring by contract research organization staff, other auditors, and even by regulatory inspectors could also be enabled by secure technologic solutions.^30,31^

We should acknowledge, however, that there are challenges to the increased use of such technologies in clinical trials, especially related to cybersecurity. It is also important to ensure that the increased use of technology does not have the unintended consequence of excluding individuals who are unable or unwilling to access that technology, such as the elderly and disadvantaged. Moreover, the increased use of technology may be both an opportunity and a threat to increasing clinical trial participation by people in low- and middle-income countries where access to mobile devices may be relatively good but other infrastructure less so.

### Cutting the Clutter

Eliminating unnecessary complexity and bureaucracy from clinical trials could help reduce the cost and time required to answer research questions.^32,33^

Reducing the verbosity and complexity of the informed consent form is long overdue.^34,35^ In case patients who are enrolled in ongoing studies need to have their consent reobtained, the updated informed consent forms should not repeat information already presented in the initial consent documents, and the application of eConsent technologies provides an efficient source of this revised information and an effective audit of review and signature.

Each data item that is collected in clinical trials generates burden and cost (data entry, multiple levels of checks, source data verification, and query generation and resolution). Despite this, a significant proportion of the data collected during cancer trials may never be used; for example, a Canadian study found that only 18% of data elements collected during clinical trials were reported in future publications,^36^ although a part of such unpublished data may still have been used effectively.

Whereas the collection of research biopsies may enable useful future correlative science, their immediate utility often remains uncertain.^37^ A strong scientific rationale for collecting tissue should be defined and ethical issues should be carefully considered before making them mandatory in cancer trials.^38^ Alternate specimen types, such as liquid biopsies, for enrollment or monitoring purposes should be considered when and where appropriate. The process of submitting diagnostic blocks and other biosamples should be streamlined and standardized,^39^ and we should aim to collect only the essential data and samples required to answer the predefined objectives of the ongoing study, allowing the option to collect more data and samples from interested patients/sites for subsequent translational research.

Published and reusable standards, rather than just templates, of trial charters, biospecimen collection protocols, and toxicity management guidelines could help reduce paperwork and duplication of effort. Attempts at the standardization of trial methodology should be encouraged and adopted.^40^ A greater ease of administration for ethical review across institutions, which would allow for a single ethics board to be designated lead for multiple centers within a region, country, or even continent, should become standard.

Regulators, such as the US Food and Drug Administration^41^; health systems, like the United Kingdom National Health Service^42^; professional oncology societies, such as the European Society of Medical Oncology and the American Society of Clinical Oncology^30,43^; academicians; and industry should continue to make efforts to streamline clinical research and reduce the burden of paperwork.^44^ It is a challenge for principal investigators to sign off and act as guarantor for electronic case record forms and multiple serious adverse event reports; it is important that investigator oversight is not diluted by collecting ever-increasing volumes of data for which they are not easily equipped to vouch and certify.

### Speedier Approvals and Permissions to Launch Clinical Trials

Regulators and stakeholders involved with planning and executing cancer studies should carefully analyze and adopt best practices where possible from some large COVID-19 trials, like RECOVERY (EudraCT 2020-001113-21), DisCoVeRy (ClinicalTrials.gov identifier: NCT04315948), and SOLIDARITY (ClinicalTrials.gov identifier: NCT04330690), which were designed quickly and built to greatly reduce the bureaucratic burden on participating sites, with rapid startup, simplified requirements for recording consent, collection of only essential data, and ease and flexibility in methods of data entry, which enabled a remarkably early first readout of efficacy.^45^

### Patient Centricity

With numerous societal changes underway, this is also an opportunity for a step change in patient involvement in clinical trials. Much progress has been made with patient and public involvement in clinical trials, grant applications, and trial oversight groups. Still, there is scope for deeper engagement with patient and public involvement groups to set the global oncology research agenda. Such a relationship must be transparent and integral to study design and conduct and not merely superficial. Many COVID-19 trials had broad entry criteria, and there is now an opportunity similarly to broaden eligibility criteria^46,47^ and to recruit an ethnically diverse population in cancer trials.^48^

The COVID-19 pandemic has given a boost to the emerging concept of the virtual or decentralized trial, which is a siteless study in which patient recruitment is done via Web-based methods that involve social media, patient portal and telemedicine applications, informed consent via remote electronic document access, review and signature, some trial activities done via video conference, physical examination done via remote visit or in-home nurse visit, laboratory specimen collection done by local clinics or in-home phlebotomist visit or patient service draw centers, data collection via digital health devices or ePROs, shipping of drugs to the patient’s home, and outcomes collected by remote methods using digital tools.^49^ A fully virtual trial is not feasible for most cancer studies, given the need for detailed and often delicate discussions, especially at the time of informed consent^50^; intravenous drug administrations; medical imaging; and toxicity surveillance. However, decentralizing some elements when appropriate could make conventional trials more efficient, potentially reducing patient burden and consequential clinical trial dropout and optimizing health care resource utilization. These hybrid trials would be located on a spectrum, with interventional clinical trials at one end and pragmatic or real-world studies at the other.^51^ A careful review of the number of mandatory visits to the hospital should be performed and, where possible, reduced.^8^ Social media platforms should be used to share results of clinical trials in innovative and patient-centered ways, including lay-language summaries and intuitive data visualization.

### Future Perspectives

The coming era of more patient-friendly clinical trials will require changes to the way cancer services are currently organized and delivered, with the aim of reducing the number of times the patient and contract research organization staff need to visit the site. Home visits by specialized nurses and phlebotomists, delivery of certain medications to patients’ homes, and precise and timely communication with family physicians or local clinics to perform laboratory testing will likely improve the patient experience. It will, however, require careful coordination by the sites, which could be challenging in areas with limited capacity or resources. We must also be careful to ensure that any changes we adopt should not result in the systematic exclusion of potential patient pools based on geographic location, rural versus urban settings, access to digital technology, educational level, ethnicity, ability, or age.^52,53^

In conclusion, the pandemic has revealed certain limitations in the current models of cancer care and the traditional conservative approach to cancer research.^54-56^ Consequently, the move toward patient centricity has accelerated, with increasing use of easily accessible and comprehensible technology, such as video, mobile phones, apps, telemedicine, and wearable devices. It has also created an opportune moment to reflect on past practices and fine tune the technologies, policies, and methodologies that we adopt in future cancer studies to enable us to develop better medicines for our patients in a faster and more efficient manner.

## References

[1] Wouters OJ McKee M Luyten J Estimated research and development investment needed to bring a new medicine to market, 2009-2018 JAMA 323 844 853 2020 32125404 10.1001/jama.2020.1166 PMC7054832

[2] Jardim DL Schwaederle M Hong DS et al An appraisal of drug development timelines in the era of precision oncology Oncotarget 7 53037 53046 2016 27419632 10.18632/oncotarget.10588 PMC5288167

[3] Steensma DP Kantarjian HM Impact of cancer research bureaucracy on innovation, costs, and patient care J Clin Oncol 32 376 378 2014 24395852 10.1200/JCO.2013.54.2548

[4] Ioannidis JP Why most clinical research is not useful PLoS Med 13 e1002049 2016 27328301 10.1371/journal.pmed.1002049 PMC4915619

[5] Saini KS de Las Heras B de Castro J et al Effect of the COVID-19 pandemic on cancer treatment and research Lancet Haematol 7 e432 e435 2020 32339482 10.1016/S2352-3026(20)30123-X PMC7195053

[6] Unger JM Blanke CD LeBlanc M et al Association of the coronavirus disease 2019 (COVID-19) outbreak with enrollment in cancer clinical trials JAMA Netw Open 3 e2010651 2020 32478845 10.1001/jamanetworkopen.2020.10651 PMC7265094

[7] Borno HT Small EJ Does the COVID-19 outbreak identify a broader need for an urgent transformation of cancer clinical trials research? Contemp Clin Trials 92 105997 2020 32272172 10.1016/j.cct.2020.105997 PMC7194679

[8] Doherty GJ Goksu M de Paula BHR Rethinking cancer clinical trials for COVID-19 and beyond Nat Can 1 568 572 2020 10.1038/s43018-020-0083-x PMC7258610 35121973

[9] Colbert LE Kouzy R Abi Jaoude J et al Cancer research after COVID-19: Where do we go from here? Cancer Cell 37 637 638 2020 32396859 10.1016/j.ccell.2020.04.003 PMC7212941

[10] US Centers for Medicare & Medicaid Services Medicare telemedicine health care provider fact sheet https://www.cms.gov/newsroom/fact-sheets/medicare-telemedicine-health-care-provider-fact-sheet

[11] National Health Service COVID-19 Information Governance advice for staff working in health and care organisations https://www.nhsx.nhs.uk/covid-19-response/data-and-information-governance/information-governance/covid-19-information-governance-advice-health-and-care-professionals/

[12] Ross C ‘I can’t imagine going back’: Medicare leader calls for expanded telehealth access after Covid-19 STAT, June 9, 2020:https://www.statnews.com/2020/06/09/seema-verma-telehealth-access-covid19/

[13] Shachar C Engel J Elwyn G Implications for telehealth in a postpandemic future: Regulatory and privacy issues JAMA 323 2375 2376 2020 32421170 10.1001/jama.2020.7943

[14] US Food and Drug Administration Dr. Hahn on COVID-19 [YouTube video]. https://www.youtube.com/watch?v=vM1Dq7muX50

[15] Welch BM Marshall E Qanungo S et al Teleconsent: A novel approach to obtain informed consent for research Contemp Clin Trials Commun 3 74 79 2016 27822565 10.1016/j.conctc.2016.03.002 PMC5096381

[16] Sirintrapun SJ Lopez AM Telemedicine in cancer care Am Soc Clin Oncol Educ Book 38 540 545 2018 30231354 10.1200/EDBK_200141

[17] Subbiah IM Hamilton E Knoll M et al A big world made small: Using social media to optimize patient care Am Soc Clin Oncol Educ Book 39 e212 e218 2019 31099651 10.1200/EDBK_246643

[18] Hollander JE Carr BG Virtually perfect? Telemedicine for Covid-19 N Engl J Med 382 1679 1681 2020 32160451 10.1056/NEJMp2003539

[19] Ohannessian R Duong TA Odone A Global telemedicine implementation and integration within health systems to fight the COVID-19 pandemic: A call to action JMIR Public Health Surveill 6 e18810 2020 32238336 10.2196/18810 PMC7124951

[20] Pell R Oien K Robinson M et al The use of digital pathology and image analysis in clinical trials J Pathol Clin Res 5 81 90 2019 30767396 10.1002/cjp2.127 PMC6463857

[21] Chauvie S De Maggi A Baralis I et al Artificial intelligence and radiomics enhance the positive predictive value of digital chest tomosynthesis for lung cancer detection within SOS clinical trial Eur Radiol 30 4134 4140 2020 32166491 10.1007/s00330-020-06783-z

[22] Beck JT Rammage M Jackson GP et al Artificial intelligence tool for optimizing eligibility screening for clinical trials in a large community cancer center JCO Clin Cancer Inform 4 50 59 2020 31977254 10.1200/CCI.19.00079

[23] Woo M An AI boost for clinical trials Nature 573 S100 S102 2019 31554996 10.1038/d41586-019-02871-3

[24] Jain NM Culley A Knoop T et al Conceptual framework to support clinical trial optimization and end-to-end enrollment workflow JCO Clin Cancer Inform 3 1 10 2019 10.1200/CCI.19.00033 PMC6873934 31225983

[25] Kim J Campbell AS Wang J Wearable non-invasive epidermal glucose sensors: A review Talanta 177 163 170 2018 29108571 10.1016/j.talanta.2017.08.077

[26] Chételat O Ferrario D Proença M et al Clinical validation of LTMS-S: A wearable system for vital signs monitoring Conf Proc IEEE Eng Med Biol Soc 2015 3125 3128 2015 10.1109/EMBC.2015.7319054 26736954

[27] Calvert M Kyte D Mercieca-Bebber R et al Guidelines for inclusion of patient-reported outcomes in clinical trial protocols: The SPIRIT-PRO extension JAMA 319 483 494 2018 29411037 10.1001/jama.2017.21903

[28] Paulsen A Overgaard S Lauritsen JM Quality of data entry using single entry, double entry and automated forms processing: An example based on a study of patient-reported outcomes PLoS One 7 e35087 2012 22493733 10.1371/journal.pone.0035087 PMC3320865

[29] Cox SM Lane A Volchenboum SL Use of wearable, mobile, and sensor technology in cancer clinical trials JCO Clin Cancer Inform 10.1200/CCI.17.00147 30652590

[30] Waterhouse DM Harvey RD Hurley P et al Early impact of COVID-19 on the conduct of oncology clinical trials and long-term opportunities for transformation: Findings from an American Society of Clinical Oncology survey JCO Oncol Pract 16 417 421 2020 32396491 10.1200/OP.20.00275

[31] European Medicines Agency Guidance on remote GCP inspections during the COVID19 pandemic https://www.ema.europa.eu/en/documents/regulatory-procedural-guideline/guidance-remote-gcp-inspections-during-covid-19-pandemic_en.pdf

[32] Gribben J Macintyre E Sonneveld P et al Reducing bureaucracy in clinical research: A call for action HemaSphere 4 e352 2020 32309789 10.1097/HS9.0000000000000352 PMC7162089

[33] Duley L Gillman A Duggan M et al What are the main inefficiencies in trial conduct: A survey of UKCRC registered clinical trials units in the UK Trials 19 15 2018 29310685 10.1186/s13063-017-2378-5 PMC5759880

[34] Duong Q Mandrekar SJ Winham SJ et al Understanding verbosity: Funding source and the length of consent forms for cancer clinical trials J Cancer Educ10.1007/s13187-020-01757-7 [epub ahead of print on May 8, 2020] 10.1007/s13187-020-01757-7 PMC7648720 32385740

[35] Corneli A Namey E Mueller MP et al Evidence-based strategies for shortening informed consent forms in clinical research J Empir Res Hum Res Ethics 12 14 25 2017 28078953 10.1177/1556264616682550 PMC5821132

[36] O’Leary E Seow H Julian J et al Data collection in cancer clinical trials: Too much of a good thing? Clin Trials 10 624 632 2013 23785066 10.1177/1740774513491337

[37] Sweis RF Drazer MW Ratain MJ Analysis of impact of post-treatment biopsies in phase I clinical trials J Clin Oncol 34 369 374 2016 26668350 10.1200/JCO.2015.63.6126 PMC4872035

[38] Levit LA Peppercorn JM Tam AL et al Ethical framework for including research biopsies in oncology clinical trials: American Society of Clinical Oncology research statement J Clin Oncol 37 2368 2377 2019 31343905 10.1200/JCO.19.01479

[39] Makhlouf H Watson MA Lankes HA et al Toward improving practices for submission of diagnostic tissue blocks for National Cancer Institute clinical trials Am J Clin Pathol 153 149 155 2020 31613330 10.1093/ajcp/aqz141 PMC7169836

[40] Blagden SP Billingham L Brown LC et al Effective delivery of complex innovative design (CID) cancer trials: A consensus statement Br J Cancer 122 473 482 2020 31907370 10.1038/s41416-019-0653-9 PMC7028941

[41] US Food and Drug Administration Evaluating the Paperwork Reduction Act Part II: Are Burdens Being Reduced? https://www.fda.gov/news-events/congressional-testimony/evaluating-paperwork-reduction-act-part-ii-are-burdens-being-reduced

[42] National Health Service 12 actions to support and apply research in the NHS https://www.england.nhs.uk/wp-content/uploads/2018/05/12-actions-to-support-and-apply-research-in-the-nhs.pdf

[43] Perez-Gracia JL Awada A Calvo E et al ESMO Clinical Research Observatory (ECRO): Improving the efficiency of clinical research through rationalisation of bureaucracy ESMO Open 5 e000662 2020 32393574 10.1136/esmoopen-2019-000662 PMC7223268

[44] Kantarjian HM Prat F Steensma DP et al Cancer research in the United States: A critical review of current status and proposal for alternative models Cancer 124 2881 2889 2018 29757456 10.1002/cncr.31522

[45] Horby P Lim WS Emberson J et al Effect of dexamethasone in hospitalized patients with COVID-19: Preliminary report medRxiv 10.1101/2020.06.22.20137273 [epub ahead of print on June 22, 2020]

[46] Beaver JA Ison G Pazdur R Reevaluating eligibility criteria: Balancing patient protection and participation in oncology trials N Engl J Med 376 1504 1505 2017 28423289 10.1056/NEJMp1615879

[47] Kim ES Bruinooge SS Roberts S et al Broadening eligibility criteria to make clinical trials more representative: American Society of Clinical Oncology and Friends of Cancer Research joint research statement J Clin Oncol 35 3737 3744 2017 28968170 10.1200/JCO.2017.73.7916 PMC5692724

[48] Tabriz AA Fleming PJ Shin Y et al Challenges and opportunities using online portals to recruit diverse patients to behavioral trials J Am Med Inform Assoc 26 1637 1644 2019 31532482 10.1093/jamia/ocz157 PMC6857600

[49] Ali Z Zibert JR Thomsen SF Virtual clinical trials: Perspectives in dermatology Dermatology 236 375 382 2020 32126560 10.1159/000506418

[50] Wolf I Waissengrin B Pelles S Breaking bad news via telemedicine: A new challenge at times of an epidemic Oncologist 25 e879 e880 2020 32304624 10.1634/theoncologist.2020-0284 PMC7288637

[51] Simon GE Platt R Hernandez AF Evidence from pragmatic trials during routine care: Slouching toward a learning health system N Engl J Med 382 1488 1491 2020 32294344 10.1056/NEJMp1915448

[52] Bhatt S Evans J Gupta S Barriers to scale of digital health systems for cancer care and control in last-mile settings J Glob Oncol 10.1200/JGO.2016.007179 10.1200/JGO.2016.007179 PMC6180787 30241157

[53] Romano MF Sardella MV Alboni F et al Is the digital divide an obstacle to e-health? An analysis of the situation in Europe and in Italy Telemed J E Health 21 24 35 2015 25495564 10.1089/tmj.2014.0010

[54] Kotecki N Penel N Awada A How to emerge from the conservatism in clinical research methodology? Curr Opin Oncol 29 400 404 2017 28704211 10.1097/CCO.0000000000000399

[55] de Miguel M Doger B Boni V et al Increased vulnerability of clinical research units during the COVID-19 crisis and their protection Cancer10.1002/cncr.32980 [epub ahead of print of June 2, 2020] PMC7300769 32557596

[56] Gyawali B Poudyal BS Eisenhauer EA Covid-19 pandemic: An opportunity to reduce and eliminate low-value practices in oncology? JAMA Oncol10.1001/jamaoncol.2020.2404 [epub ahead of print on July 2, 2020] 10.1001/jamaoncol.2020.2404 32614374

